# Vitamin D and donepezil intervention in ovariectomized rats: modulating neuroinflammation in the aging brain

**DOI:** 10.1002/alz.091766

**Published:** 2025-01-03

**Authors:** Lucineia Danielski, Eduarda Behenck Medeiros, Gabriela Piovesan Fenilli, Gabriel Casagrande Zabot, Adrielly Vargas, Amanda L Boaventura, Mariana Cardoso Lumertz, Franciane Bobinski, Vijayasree V Giridharan, Tatiana Barichello, Josiane Budni

**Affiliations:** ^1^ The University of Texas Health Science Center at Houston, Houston, TX USA; ^2^ Universidade do Extremo Sul Catarinense, Criciuma, SC Brazil; ^3^ University of Southern Santa Catarina (UNESC), Criciuma, SC Brazil; ^4^ Laboratory of Experimental Neurology, Graduate Program in Health Sciences (PPGCS), University of Southern Santa Catarina (UNESC), Criciuma, SC Brazil; ^5^ Universidade do Sul de Santa Catarina, Palhoca, SC Brazil

## Abstract

**Background:**

Aging is a natural, irreversible process that can be successful or pathological, resulting in chronic degenerative diseases such as Alzheimer’s disease. Low levels of estrogen characterize menopause. Research reveals that the lack of these hormones may be related to dementia and that vitamin D (vit D), when supplemented, has a neuroprotective and neuromodulator effect.

**Method:**

60‐ or 120‐day‐old Wistar rats were subjected to ovariectomy (OVX) for either 1 or 8 months, treatment with oral administration of Vit D (42 or 420 IU/kg) and/or DONE (1 mg/kg) spanned 21 days. On the 22nd day of the protocol, 24 hours post the final administration, the prefrontal cortex and hippocampus were dissected for cytokine analysis, including interleukin 10 (IL‐10), IL‐1 β, and tumor necrosis factor α (TNF‐ α), conducted through enzyme immunoassay.

**Result:**

In 60‐day‐old rats undergoing one month of ovariectomy (OVX), elevated IL‐1β and TNF‐α levels were observed in the brain. The optimal dose for mitigating these effects was found to be vitamin D (Vit D) at 420 IU/kg, either alone or in combination with DONE. This dosage successfully reversed the increased IL‐1β levels in the frontal cortex and normalized the elevated TNF‐α levels in the hippocampus. Similarly, in rats subjected to OVX for eight months, the most effective dose for reversing increased IL‐1β levels in the frontal cortex and hippocampus was Vit D at 420 IU/kg. However, in this protocol, TNF‐α levels remained unchanged, emphasizing the selective impact of this particular dosage. Additionally, the optimal dose for modulating IL‐10 levels in the frontal cortex, particularly in the context of increased IL‐10 following OVX, was Vit D at 420 IU/kg.

**Conclusion:**

The administration of vitamin D either alone or in combination with DONE (a specific treatment) demonstrated favorable outcomes in the rats, effectively mitigating the inflammatory response.

